# Optimization, Ex-Vivo Penetration and In-Vivo Evaluation of Pegylated Nanostructured Lipid Carriers of Olmesartan Medoxomil to Enhance Transdermal Delivery

**DOI:** 10.34172/apb.46458

**Published:** 2026-05-19

**Authors:** Rofida Albash, Aly A. Abdelbary, Hanan Refai, Mohamed A. El-Nabarawi

**Affiliations:** ^1^Department of Pharmaceutics, College of Pharmaceutical Sciences and Drug Manufacturing, Misr University for Science and Technology, Giza, Egypt; ^2^Department of Pharmaceutics and Industrial Pharmacy, Faculty of Pharmacy, Cairo University, Cairo, Egypt; ^3^Department of Pharmaceutics and Industrial Pharmacy, Faculty of Pharmacy, University of Hertfordshire, New Capital, Egypt

**Keywords:** Antihypertensive study, Factorial design, Olmesartan medoxomil, PEGylated nanostructured lipid carriers, Shed snake skin, Transdermal delivery

## Abstract

**Purpose:**

Olmesartan medoxomil (OLM), a poorly water soluble hypertension medication with 26% oral bioavailability, was encapsulated in PEGylated nanostructured lipid carriers (PNLCs) for transdermal distribution.

**Methods:**

Tween 80 or sodium deoxycholate stabilized the PNLCs, which were made up of liquid lipid including a mixture of medium chain triglycerides (labrasol and labrafil) and solid lipid (compritol and tristearin). Hot melt emulsification, high-speed stirring, and ultrasonication were used to create PNLCs. To distinguish the effects of formulation independent variables on entrapment efficiency %, particle size, polydispersity index, zeta potential, and amount of drug released after 6 hours (Q6h), a 24 complete factorial design using Design Expert® software was created.

**Results:**

For additional research, the formula (PNLC16) with the best requirements was chosen. When compared to OLM solution, PNLC16 showed higher permeation ex vivo permeation using both shed snake and rat skin. Histopathological analysis demonstrated that the topically administered PNLCs were safe. Pharmacodynamic research further demonstrated PNLC16’s superiority over commercial oral pills in terms of long-lasting effects.

**Conclusion:**

Accordingly, the results showed that PNLC16 would be a viable vehicle for OLM transdermal delivery.

## Introduction

 Because of its ability to effectively control the renin-angiotensin-aldosterone system, angiotensin II receptor antagonists—such as the hydrophobic medication olmesartan medoxomil (OLM)—have attracted interest as antihypertensive medications.^1^ OLM is a hydrophobic ester prodrug of olmesartan that is absorbed from the gastrointestinal system and converted to the active olmesartan by ester hydrolysis. However, because of its first-pass metabolism and poor water solubility, OLM has a very low oral bioavailability of 26%.^2^

 One possible solution to oral deficiencies is transdermal administration. Additionally, it avoids the first pass effect; if any side effects develop, therapy could be stopped by removing the transdermal drug delivery system from the skin.^3^ Getting past the stratum corneum (SC) is the main challenge in transdermal medication administration. To passively diffuse through the drug should be nonionized, with molecular weight < 400–500 Da, lipophilic with a log P ranging from 1 up to a maximum of 4, with a melting point less than 200°C and provided in a modest dose.^4^

 Convenient methods, such as using lipid nanocarriers like solid lipid nanoparticles (SLNs), could be used to increase skin penetration. When compared to other colloidal systems, SLNs have a few advantages, such as the capacity to be manufactured on a large scale and the ability to be prepared without the need of an organic solvent. SLN is made up of pure lipids, however when solid lipids transition to low energy shapes, they form perfect crystalline structures that cause the encapsulated medicine to be expelled during storage. As a result, during storage, the amount of encapsulated medication and the rates of drug release from SLNs may vary.^5^

 In order to overcome these issues, a combination of solid and liquid lipids was used to create nanostructured lipid carriers, or NLCs.^6^ NLCs have higher loading capacity and lower drug expulsion on storage compared to SLNs.^7^ The skin accepts NLCs well, and scaling up is simple. Additionally, a large surface area is available for medication absorption through the skin surface due to the smaller particle size. Furthermore, lipids and surfactants (SAAs), which are components of NLCs, may act as permeation enhancers to promote drug entry into the lipid bilayers of SC.^8^

 PEGylated nanostructured lipid carriers (PNLCs) were developed for transdermal administration of OLM in the current investigation. PNLCs were prepared using two different forms of liquid lipids, labrafil and labrasol, which are made of PEG/glycerides with varying HLB values. These liquid lipids’ emulsifying qualities are caused by the coupling of the PEG moieties with hydrophobic molecules.^9^ Additionally, PEGylation may increase drug penetration into the skin by binding to water molecules that may improve the SC’s hydration, resulting in effective skin penetration.^10^

 To the best of our knowledge, PNLCs have not been the subject of any published studies on drug delivery for OLM. Accordingly, the main goal of this study was to create the best PNLC formulations possible for successful OLM entrapment using hot melt emulsification in conjunction with high-speed stirring and the ultra-sonication method, as well as to look into how formulation variables affect PNLC properties. The best formula was determined using Design Expert^®^ software and a 2^4^ complete factorial design. While entrapment efficiency percent (Y_1_), particle size (Y_2_), zeta potential (Y_3_), and amount of drug released after 6 hours (Q6h) (Y_4_) were chosen as dependent variables, solid lipid type (X_1_), liquid lipid type (X_2_), solid lipid to liquid lipid percentage (X_3_), and SAA type (X_4_) were examined as independent variables. The ideal PNLC was assessed for *ex vivo* penetration, stability analysis, and morphology. Additionally, pharmacokinetic and histopathological investigations were carried out on male Wistar rats.

## Materials and Methods

###  Materials

 Olmesartan medoxomil (OLM) was gifted from FAP Pharm. (Cairo, Egypt). Compritol 888 ATO^®^, Tristearin^®^, Labrafil 2125^®^ and Labrasol^®^ were obtained as a gift from Gattefosse Co. (St. Priest, France). Sodium deoxycholate (SDC) and cellulose membrane were purchased from Sigma Aldrich Chemical Co. (St. Louis, USA). Tween 80 (T80), potassium dihydrogen phosphate, and dipotassium hydrogen phosphate were obtained from El-Nasr Pharmaceutical Chemicals Co. (Cairo, Egypt). Methyl prednisolone acetate (MPA) Depo Medrol^®^ Pfizer (Cairo, Egypt). Ethanol 95%, methanol, acetonitrile and triethylamine HPLC grade were provided by Merck Co. (Darmstadt, Germany). Angiosartan^®^ 10 mg oral tablets, indicated in the text as the market tablets, were gained from Chemipharm Co. (Cairo, Egypt).

###  Methods

####  Preparation of PNLCs

 Using a 2^4^ complete factorial design and Design Expert® software, PNLCs were made by hot melt emulsification combined with high-speed stirring and ultrasonication (Table 1). To create a clear oil phase, OLM (25 mg) was combined with liquid and solid lipids and heated while being stirred to a temperature ten degrees higher than the melting point of the solid lipid.^11^ The total amount of solid lipid and liquid lipid was 1% (w/w). Double-distilled water (10 mL) with 1% (w/w) SAA added as a stabilizer made up the aqueous phase. The lipid and aqueous phases were heated separately for 10 min at the same temperature. Then the aqueous phase was added dropwise to the lipid phase and mixed at 1500 rpm using high-speed magnetic stirrer for 10 min. The obtained pre-emulsion was then treated by probe sonicator (3 min, 3s on/off and 50% voltage efficiency) (JY-92-II, Xinzhi, China) for 5 min. The final volume of the dispersion was 10 mL. The obtained dispersion was stored overnight at 4°C and then subjected to further characterization.

**Table 1:** Full factorial design (2^4^) used for optimization of PNLC formulations.

**Factors (Independent variables)**	**Levels**	
X_1_: Solid lipid type	Compritol	GTS
X_2_: Liquid lipid type	Labrasol	Labrafil
X_3_: Solid lipid: Liquid lipid	70:30	50:50
X_4_: SAA type	T80	SDC
**Responses (Dependent variables)**	**Desirability constraints**	
Y_1_: EE%	Maximize	
Y_2_: PS (nm)	Minimize	
Y_3_: ZP (mV)	Maximize (as absolute value)	
Y_4_: Amount of drug released after Q6h (%)	Maximize	

Abbreviations: PNLC, PEGylated nanostructured lipid carriers; GTS, tristearin; SAA, surfactant; T80, Tween 80; SDC, Sodium deoxycholate; EE%, entrapment efficiency percentage; PS, particle size; and ZP, Zeta potential.

###  In-Vitro Studies of OLM -PNLCs

####  Entrapment Efficiency Percent (EE%)

 A cooling centrifuge (Sigma 3K 30, GmbH, Germany) was used to centrifuge the produced formulae for one hour at 4°C and 20,000 rpm. Methanol was then used to break down the sediment, and a UV-Vis spectrophotometer (Shimadzu UV 1650, Japan) was used to detect the sediment’s λ_max_ at 257 nm. As previously mentioned in the literature, the direct technique was used to determine entrapment.^12^

####  Determination of Particle Size (PS), Polydispersity Index (PDI), and Zeta Potential (ZP)

 A Zetasizer (Malvern, England) was used to measure the mean PS, PDI, and ZP for the prepared equations. The PS and PDI were determined by evaluating the variations in light scattering. By monitoring the particles’ electrophoretic mobility in the electrical field, the ZP was assessed.^3^

####  Determination of the Amount of Drug Released After 6h (Q6h) 

 The USP dissolving apparatus (Pharma Test, Germany) was used to measure Q6h from the produced formulae for six hours at 37°C. In plastic cylindrical tubes with a release area of 3.14 cm^2^, two milliliters of PNLC formulations containing 5 mg of OLM each were put in. One end of the tubes was covered with a cellulose membrane, and the other end was attached to the shaft of the USP dissolve apparatus rather than the baskets. The cylindrical tubes were submerged in 50 mL composed of 30:20% v/v (PBS pH of 7.4: ethanol mixture, total volume 50 mL).^13^ The sink condition was maintained. Aliquots were withdrawn at 1, 2, 3, 4, 5 and 6 h. OLM in samples was analyzed by UV spectrophotometer at λ_max_ 257 nm.

###  Studying the Significance of Formulation Variables by 2^4^ Factorial Design

 A useful technique for precisely delivering the best results is design of experimentation (DOE), which is recognized to reduce the number of trials while adequately screening the process parameters. Additionally, it ensures the procedures’ quality in terms of mathematical equations. Only after determining the ideal values of its variables for a high-quality product can a process be accredited. One useful tool for determining the ideal values of variables is desirability. To ascertain the impact of variables on creating PNLCs to increase the permeability of OLM, a 2^4^ complete factorial design was employed. Solid lipid type (X_1_), liquid lipid type (X_2_), solid lipid: liquid lipid % (X_3_), and SAA type (X_4_) were selected as independent variables, while EE% (Y_1_), PS (Y_2_), ZP (Y_3_), and Q6h (Y_4_) were arranged as dependent variables (Table 1).

###  Optimization of OLM PNLCs

 The desirability tool, which allowed for simultaneous viewing of all responses, served as the basis for choosing the best formula. The goal of the optimization step was to obtain a formula with the highest EE%, ZP (as an absolute number), and Q6h, and the lowest PS. The solution that had an attractiveness estimate of around one was chosen. The ideal formula was created, described, and contrasted with the anticipated results to validate the model’s effectiveness

###  Transmission Electron Microscopy (TEM)

 A transmission electron microscope was used to observe the external shape of the optimum formula under magnification power 2000 × (Joel JEM 1230, Japan). A drop of the particle dispersion was placed onto a carbon coated copper grid then stained and after that photographed.^12^

###  Differential Scanning Calorimetry (DSC)

 Differential scanning calorimetry (DSC-60, Shimadzu, Japan) calibrated with purified indium at a temperature range of 10-250°C and a scanning rate of 5°C/min under inert nitrogen flow was used to thermally investigate OLM, GTS, labrafil, SDC, a physical mixture of OLM with PNLC constituents, and the ideal PNLC.^13^

###  Stability Study

 For ninety days, the ideal mixture was kept between 4°C and 25°C. Samples from each formulation were withdrawn at 0, 45 and 90 days. Stability was assessed by applying comparative assessment of the measurements of freshly prepared and stored samples.^14^ PNLCs’ EE%, PS, PDI, ZP, and Q6h were measured as previously shown. Using SPSS® 22.0, the Student’s t-test was used to determine statistical significance. At *P* ≤ 0.05, the change was deemed significant.

###  Ex-Vivo Permeation Via Shed Snake Skin and Rat Skin 

 It was assessed how well the OLM suspension and the ideal PNLC penetrated the skins of rats and shed snakes. The skin of a shed snake was donated by the Zoo in Giza, Egypt. Animals with cervical dislocation had their rat skin removed. The dorsal skin was used in place of the cellulose membrane in the test assembly, which was identical to that used in the in-vitro release. All measurements were made in triplicate ± SD, and samples were taken from the receiving compartment at 2, 4, 8, 10, and 12 hours before being subjected to HPLC analysis. The enhancement ratio (ER) and the permeation flux (Jmax) at 12 hours were computed.^15^ Statistical significance was analyzed by Student’s t-test using SPSS^®^ software 22.0. The difference at *P* ≤ 0.05 was regarded significant.

###  In-Vivo Studies

 For the in-vivo tests, thirty-three male Wistar rats weighing between 150 and 200 g took part. The study design was authorized by the ethical committee of the Department of Pharmaceutics and Industrial Pharmacy, Cairo University (reference number = (PI) 1867), which obeys the European Community guidelines. Cages with a temperature of 22°C and a humidity of 55% were used to house the animals. Water and rodents’ chow were supplied. The animals were put in a dark: light cycle of 12 h each.

 Rats were kept for seven days in order to adjust. Twenty-four animals were engaged in the pharmacodynamic investigation, while nine animals were recruited in the histopathological examination. The rat dorsal skin was shaved before the OLM suspension and ideal PNLC formulation were applied, and bottle caps with a 4.91 cm^2^ area were used as drug containers. Furthermore, the application was incorporated non-occlusively within the medication packaging.^13,15^

###  Histopathological Study 

 Three groups of three male Wistar rats each were created from the nine total. Group one was used as a control, and groups two and three received topical applications of 1 mL of OLM suspension and the ideal PNLC formula, respectively, for 24 hours on a 4.91 cm^2^ shaved area. After the rats were killed, the treated skin was meticulously examined. Skin samples were dehydrated and washed after being fixed in 10% formal saline for 24 hours. Following xylene refinement, the specimens were placed in blocks of paraffin wax and kept at 56°C for an additional 24 hours. The blocks of paraffin beeswax were then sectioned at 4 mm using a sledge microtome (Rotary Leica RM2245, England). The specimens were deparaffinized and stained with hematoxylin and eosin stains for examination using light microscopy (Axiostar plus, USA).^15^

###  Pharmacodynamic Antihypertensive Study

 Based on cuff tail assessment, the evaluation was carried out using the animal tail non-invasive blood pressure method. Four groups of six male Wistar rats each (groups A through D) were created from the twenty-four males.

 For minimizing tail-cuff measurement variability, animals were acclimated to the restraint device and the measurement room for 5 consecutive days prior to baseline recording (10–15 min per day) to reduce stress-related BP elevation. All measurements were performed at a controlled ambient temperature of 37 ± 1 °C and at the same time of day to minimize circadian variability. For each time point, we recorded 4 consecutive readings per animal, discarded the first reading, and averaged the remaining 3 values to compute the reported systolic and diastolic BP. Group A served as the standard control. Methyl prednisolone acetate (MPA) (20 mg/kg/week) was injected subcutaneously into the remaining groups (groups B to D) for two weeks in order to cause hypertension. Group B was used as a control group for hypertension. The rats in Group C were given market tablets containing 10 mg of OLM (Angiosartan^®^, Egypt) that had been crushed beforehand and given to them via oral gavage. The best PNLC solution (10 mg) was applied topically to Group D. The 4.91 cm^2^ shaved abdomen region received a topically applied particle dispersion. After that, the rat was put in the restrainer, and at pre-arranged intervals of 0, 1, 2, 4, 6, 8, 24, 26, 28, and 48 hours, the blood pressure (BP) from the tail was measured.^16^ Statistical significance was analyzed by ANOVA. For multiple comparison post hoc Tukey Kramer HSD (Honestly Significant Difference) was utilized. Percent of reduction in BP from hypertensive control was calculated as mentioned in our previous work.^13^

## Results and Discussion

###  Screening of Component and Pre-Formulation Study

 In order to determine the appropriate ranges of the independent variables, a stepwise screening of the components needed to prepare PNLCs was carried out (data not shown). Solid lipid type (X_1_), liquid lipid type (X_2_), solid lipid: liquid lipid percentage (X_3_), and SAA type (X_4_) were the four criteria taken into consideration. Design Expert® software (Stat Ease, Inc., USA) was used to explore experimental results to define independently the major effects of these factors followed by analysis of variance (ANOVA) to determine the impact of each factor. It is important to note that precipitation of nanosized lipid particles caused the amount of total lipid to change from 5% to 2% and subsequently 1% (w/w). Because PNLCs were tiny and had a low lipid density, they had trouble settling when centrifuged.^17^ The two factor interaction (2FI) model was chosen, the analysis showed results in Table 2 that the adjusted R2 in each response was in feasible agreement with the projected R2 values. The is preferable to have sufficient precision with a ratio larger than 4, which was noted in every response as indicated in Table 2.

**Table 2:** Output data of the 2^4^ factorial analysis of PNLC formulations and observed values for the optimum PNLC.

**Responses**	**EE (%)**	**PS (nm)**	**ZP (mV)**	**Amount of drug released after Q6h (%)**
Adequate precision	69.85	17.81	10.80	47.29
Adjusted R^2^	0.994	0.891	0.818	0.985
Predicted R^2^	0.991	0.829	0.715	0.976
Significant factors	x_1_, x_2_, x_3,_ x_4_	x_1_, x_2_, x_3_, x_4_	x_4_	x_1_, x_2_, x_3_, x_4_
Predicted value of optimum formula (PNLC16)	41.15	340.41	-40.35	47.48
Observed value of optimum formula (PNLC16)	39.60	374.50	-44.40	48.48

Abbreviations: PNLC, PEGylated nanostructured lipid carriers; EE %, entrapment efficiency percentage; PS, particle size; PDI, polydispersity index; ZP, zeta potential. Data represented as mean ± SD (*n*= 3)

###  Factorial Design Analysis

 Only important factors are covered below, including the effects of solid lipid type, liquid lipid type, solid lipid to liquid lipid percentage, and SAA type on EE%, PS, ZP, and Q6h. All of the produced particles had a negative charge and were in the nanometer range. It is noteworthy that PDI was not significantly impacted by any of the variables (Table 3). Furthermore, no particular link was found between PDI and any of the independent variables.

 EE% = 55.53* 6.69* A 4.625 * B -2.35 * C-20.95* D -0.25* A * B -0.23* A * C-1.19 * A * D -0.65* B * C-0.43* B * D 0.38* C *D

 PS = 337.18*85.36* A48.97 * B-42.43* C -72.17* D 28.59* A * B-18.94* A * C-38.51 * A * D-11.93* B * C 6.07 B * D18.22 * C * D

 ZP = -28.37-1.34* A-1.57 * B0.17* C-6.23* D0.23*A* B-0.56* A * C-1.09 * A * D 0.29* B * C-2.52* B * D0.65 * C * D

 Q6h = 46.68-3.18* A-3.92 * B1.09* C7.18* D0.63* A * B0.06* A * C-0.35* A * D-0.72* B * C-0.21* B * D0.23* C * D

 The lack of fit was EE% 0.09, PS 0.06, PDI 0.1423, ZP 0.21, and Q6h 0.13.

**Table 3:** Experimental runs, independent variables, and measured response of the 2^4^ full factorial experimental design

	**X**_1_	**X**_2_	**X**_3_	**X**_4_	**Y**_1_	**Y**_2_		**Y**_3_	**Y**_4_
	**Solid lipid type**	**Liquid lipid type**	**SAA type**	**(Solid: liquid lipid) ** **(w/w)%**	**EE%**	**PS** **(nm)**	**PDI**	**ZP ** **(mV)**	**Amount of drug** ** released after Q6h % **
PNLC1	Compritol	Labrasol	T80	(70:30)	66.06 ± 0.10	294.1 ± 19.90	0.05 ± 0.02	-22.70 ± 0.99	45.71 ± 0.17
PNLC2	Compritol	Labrasol	SDC	(70:30)	24.72 ± 0.53	228.5 ± 10.50	0.05 ± 0.005	-31.40 ± 1.38	58.56 ± 1.16
PNLC3	Compritol	Labrasol	T80	(50:50)	62.17 ± 0.65	242.20 ± 1.70	0.07 ± 0.02	-18.80 ± 0.21	46.81 ± 0.22
PNLC4	Compritol	Labrasol	SDC	(50:50)	22.28 ± 0.25	160.90 ± 8.20	0.01 ± 0.09	-28.20 ± 10.32	64.36 ± 0.95
PNLC5	Compritol	Labrafil	T80	(70:30)	75.80 ± 0.67	324.00 ± 11.00	0.2 ± 0.04	-21.60 ± 1.28	37.38 ± 0.64
PNLC6	Compritol	Labrafil	SDC	(70:30)	38.40 ± 0.41	254.60 ± 3.60	0.09 ± 0.005	-38.00 ± 3.18	52.99 ± 1.35
PNLC7	Compritol	Labrafil	T80	(50:50)	69.33 ± 1.64	281.50 ± 5.50	0.07 ± 0.01	-23.30 ± 1.08	39.15 ± 0.53
PNLC8	Compritol	Labrafil	SDC	(50:50)	31.26 ± 0.69	228.60 ± 12.60	0.07 ± 0.02	-35.10 ± 3.02	52.05 ± 0.59
PNLC9	GTS	Labrasol	T80	(70:30)	81.56 ± 0.61	501.00 ± 49.00	0.05 ± 0.01	-18.40 ± 4.91	38.67 ± 0.51
PNLC10	GTS	Labrasol	SDC	(70:30)	39.12 ± 2.19	251.20 ± 6.50	0.05 ± 0.07	-31.60 ± 0.76	51.69 ± 0.07
PNLC11	GTS	Labrasol	T80	(50:50)	75.83 ± 0.70	428.50 ± 10.50	0.05 ± 0.02	-28.60 ± 0.58	42.37 ± 0.35
PNLC12	GTS	Labrasol	SDC	(50:50)	36.76 ± 0.08	199.20 ± 6.80	0.04 ± 0.02	-32.90 ± 2.50	55.44 ± 0.05
PNLC13	GTS	Labrafil	T80	(70:30)	94.10 ± 0.74	761.00 ± 1.00	0.04 ± 0.005	-20.40 ± 0.81	32.58 ± 0.15
PNLC14	GTS	Labrafil	SDC	(70:30)	45.74 ± 1.26	422.50 ± 19.50	0.04 ± 0.01	-44.60 ± 5.87	46.02 ± 0.94
PNLC15	GTS	Labrafil	T80	(50:50)	87.70 ± 1.59	442.50 ± 31.50	0.06 ± 0.005	-19.90 ± 0.75	33.11 ± 0.66
PNLC16	GTS	Labrafil	SDC	(50:50)	39.60 ± 1.90	374.50 ± 39.50	0.04 ± 0.01	-44.40 ± 5.38	48.48 ± 1.17

Abbreviations: PNLC, PEGylated nanostructured lipid carriers; GTS, tristearin; SAA, surfactant; T80, Tween 80; SDC, Sodium deoxycholate; EE%, entrapment efficiency percentage; PS, particle size; PDI, polydispersity index; ZP, Zeta potential. Data represented as mean ± SD (n = 3)

###  The Effect of Solid Lipid Type (X_1_)

Figure 1 (A-D) shows the graphic representation of the impact of solid lipid type (X_1_) on EE%, PS, ZP, and Q6h. Table 3 presents the findings. When compared to compritol, GTS produced noticeably (*P* < 0.0001) greater EE% readings. This could be connected to the fact that GTS has a higher affinity for OLM since it is more lipophilic than compritol.^18^ Additionally, the fact that compritol is a mixture of 13%–21% monoacylglycerols, 40%–60% diacylglycerols, and 21%–35% triacylglycerols of behenic acid may be the cause of these outcomes. Because acyl glycerol is amphiphilic with HLB, the mono and diglycerides of glyceryl behenate have surface-active properties (2–5).^19^ Compritol’s amphiphilic nature suggests that it may have dissolved OLM into the aqueous phase, lowering EE% values and, in turn, increasing the extent of drug release (Q6h) (*P* < 0.0001) in comparison to GTS.

**Figure 1:**
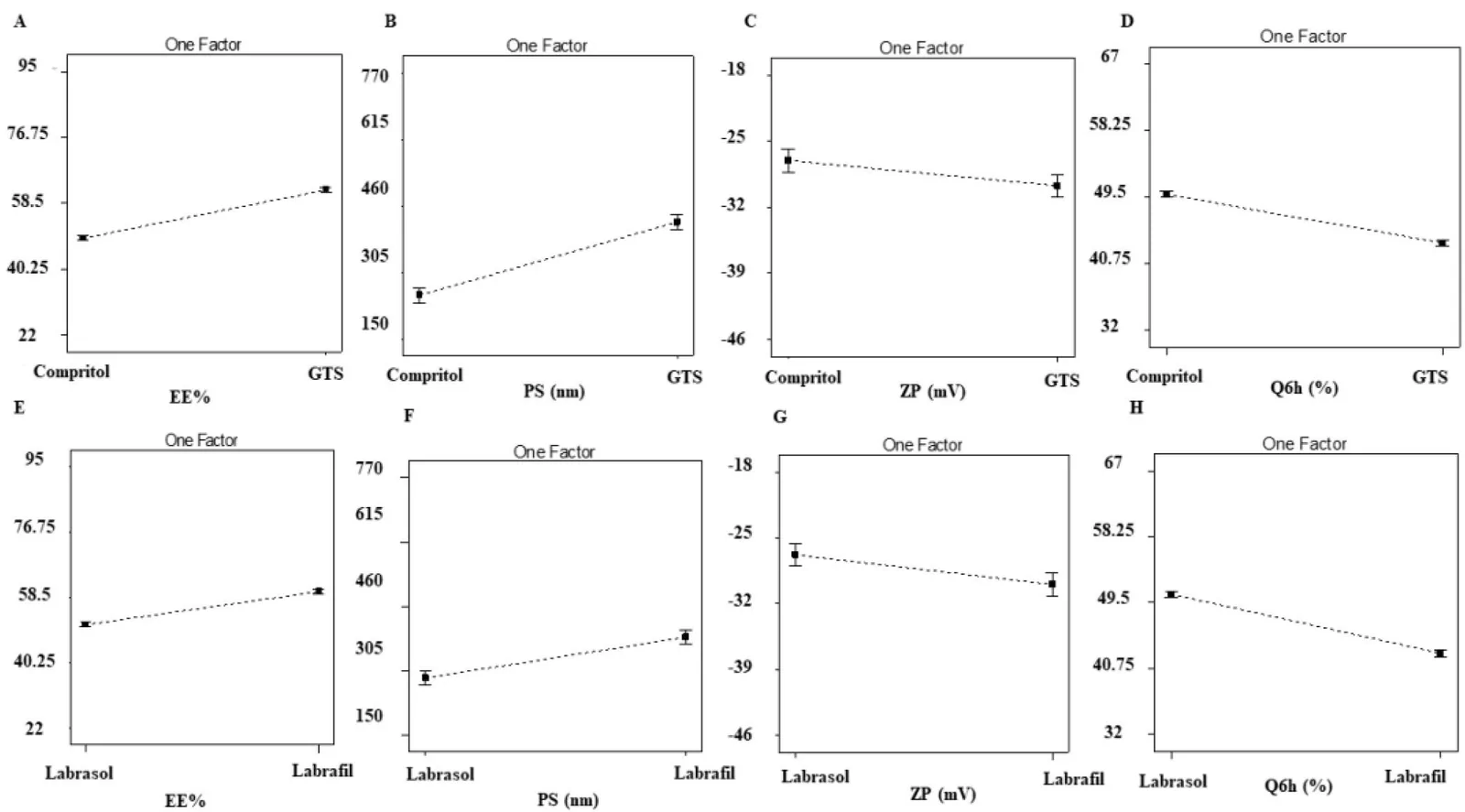
One factor plots for the effect of solid lipid type on (A) EE%, (B) PS, (C) ZP and (D) Q6h (%) and the effect of liquid lipid type on (E) EE%, (F) PS, (G) ZP and (H) Q6h (%) of OLM loaded PNLCs

 Additionally, the use of GTS resulted in a significant (*P* < 0.0001) large increase in PNLCs’ PS. The MW discrepancy between compritol (414.671 g/mol) and GTS (891.501 g/mol) may be the cause of this. Additionally, as the MW rises, the viscosity rises as well, causing PNLCs to aggregate and PS to grow.^20^ Additionally, the smaller PS that compritol produced may be explained by the fact that it functions as a nonionic emulsifier in addition to a lipid matrix, which would likely lessen particle aggregations and produce smaller PNLCs.^19^ According to the findings, PNLCs made with GTS generated noticeably (*P* = 0.03) greater absolute ZP values than compritol. This can be explained by the lipid particle matrix of GTS, which have a comparatively high concentration of ionizable fatty acids that are enough to coat the particle surface densely.

###  The Effect of Liquid Lipid Type (X2) 

Figure 1 (E-H) visually depicts the impact of liquid lipid type (X_2_) on EE%, PS, ZP, and Q6h, and Table 3 displays the findings. Compared to labrasol, labrafil yielded noticeably (*P* < 0.0001) greater EE% readings. Glycerides and free PEG units make up the mixture of both liquid lipids. Labrafil is a mixture of mono, di and triglycerides and mono and di-fatty esters of polyethylene glycol 300, which contains esters of linoleic acid and polyoxyethylene (6 PEG units).^21^ Labrasol is a blend of mono-, di-, and triglycerides as well as mono- and di-fatty esters of polyethylene glycol 400, which includes polyoxyethylene (8 PEG units) and esters of caprylic and capric acids.^22,23^ According to Wan et al, lipid nanoparticles’ PEG presence lowers their EE% values. This could explain why formulations including labrasol with eight PEG units showed lower EE% values than those containing labrafil with six PEG units.^24^ The potential solubilizing and wetting effects of PEG, which may create a hydrophilic environment around the drug and lower OLM’s affinity for the lipid core of PNLCs, may be the cause of the decrease in OLM’s EE% that results from increasing PEG concentration. Furthermore, labrafil’s lower HLB value (3.5).^25^ might have led to increased drug affinity towards PNLCs matrix. On the other hand, labrasol with higher HLB value (14).^26^ resulted in lower EE% values.

 Because PEG may have slowed down the rate of particle precipitation, which causes particle agglomeration, PS changed to lower levels as the PEG concentration in the formulations rose by employing labrasol as opposed to labrafil.^27^ Additionally, particles with higher PS may have been produced by labrafil’s increased MW compared to labrasol.^28^

 Furthermore, when compared to labrasol, labrafil generated noticeably (*P* = 0.01) greater ZP values. Lower ZP values appear to be the consequence of the hydrophilic PEG steric barrier in labrasol partially blocking the surface charge of the carboxyl groups on the particle surface.^29^

 Regarding Q6h, the findings indicate that PNLCs with labrasol discharged a considerably (*P* < 0.0001) greater amount of OLM than those with labrafil. This finding is attributed to lower EE% of the former, which resulted in increased free unentrapped drug in the external aqueous medium and accordingly higher release profiles.^30^ Another theory is that, in comparison to PNLCs made with labrafil, the smaller PS of PNLCs made with labrasol enhanced the release profile by increasing the total surface area.^31^

###  The Effect of Solid Lipid: Liquid Lipid %

 The results are shown in Table 3, and Figure 2 (A-D) graphically depicts the impact of solid lipid: liquid lipid % (X_3_) on EE%, PS, ZP, and Q6h. ANOVA results showed that changing solid lipid: liquid lipid % (X_3_) (*P* < 0.0001) from 70:30 % to 50:50 % w/w had a negative impact on EE%. Generally, all formulations containing 70% w/w solid lipid exhibited higher EE% values than those containing 50% w/w solid lipid in either type of solid lipids. This might be due to the higher viscosity of the formulations with high solid lipid content that might prevent drug leaching to the external aqueous phase, thus ensuring higher EE% values. On the other hand, by decreasing the solid lipid relative to liquid lipid content, a matrix with a larger number of pores is formed, which facilitates the escape of the drug to the external phase, resulting in lower EE%.^32^

**Figure 2:**
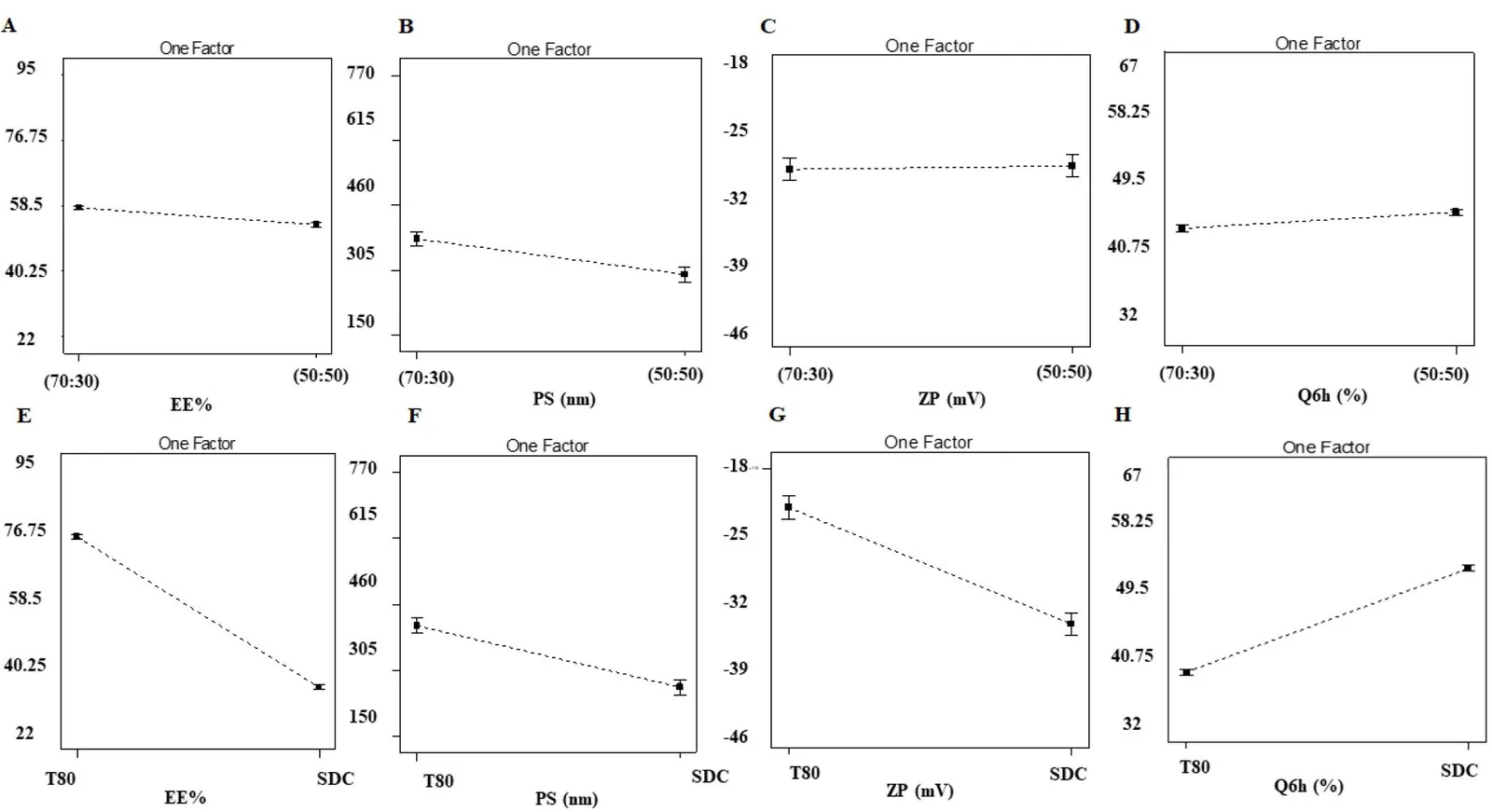
One factor plots for the effect of solid lipid: liquid lipid % on (A) EE%, (B) PS, (C) ZP and (D) Q6h (%) and the effect of SAA type on (E) EE%, (F) PS, (G) ZP and (H) Q6h (%) of OLM loaded PNLCs

 Our data for Q6h clearly demonstrated a controlled and extended release of OLM at 70% liquid lipid (*P* < 0.0001). In contrast, PNLCs with 50% liquid lipid were unable to hold onto OLM in their matrices. As previously noted by Pokharkar et al, this may be caused by pore creation, but it may also be the result of the drug’s rapid diffusion through liquid lipid, which was proposed as the release mechanism.^33^ Furthermore, a higher viscosity of the lipid matrix with higher solid lipid content likely results in more control over the released medication. The PS was substantially (*P* < 0.0001) bigger when the solid lipid to liquid lipid ratio was higher. According to Song et al, bigger particles are created when the solid-to-liquid lipid ratio increases because SAAs find it difficult to lower the surface tension of melted lipid.^34^ However, the quantity of melted solid lipid droplets drops at lower solid lipid to liquid lipid ratios, and there is enough SAA present to cover the produced nanoparticles, resulting in the creation of smaller particles. Moreover, the increase in the amount of liquid lipid tends to promote the formation of smaller particles as a result of the higher molecular mobility of the formed matrix.^35^ Further, Agrawal et al. reported that solid lipids during the solidification might form a solid lipid core in which liquid lipid is randomly distributed.^36^ In addition to being dispersed throughout the solid lipid core, which results in smaller particles, liquid lipids would be found at the outside shell of the nanoparticles when the liquid lipid percentage was higher. Thus, data demonstrate that the ideal ratio of solid to liquid lipid mixture determines the PS of PNLCs.

###  The Effect of SAA Type

 The results are shown in Table 3, and Figure 2 (E-H) graphically depicts the impact of SAA type (X4) on EE%, PS, ZP, and Q6h. In comparison to PNLCs containing SDC, T80-containing PNLCs generated considerably (*P* < 0.0001) higher EE%, larger PS, and lower absolute ZP values. It is commonly recognized that a SAA’s hydrophilicity and affinity for lipophilic medicines decrease with increasing HLB value.^37^ The HLB value of T80 is 15 and that of SDC is 23.4,^13^ which explains the greater EE% values observed for formulae containing T80. Further, as the HLB value of SAAs increases the surface free energy of the particles decreases leading to smaller particles,^38^ consequently SDC produced smaller particles compared to T80. In addition, the significant (*P* < 0.0001) decrease in PS of PNLCs by using SDC could also be attributed to its anionic nature that hindered particles’ aggregation and increased the inter-particle repulsion.^39^ Conversely, because T80 is a nonionic stabilizer, its thick hydrophobic tail enhances PS of T80-containing PNLCs, stabilizing the system through steric stabilization.^40^ The results also showed a strong link between the PS and the quantity of medication trapped in the PNLCs. Thus, another explanation for the lower PS would be the drop in EE% shown for SDC formulas^41^

 In reference to Q6h, the lower PS of SDC PNLCs particles in comparison to T80-containing PNLCs led to a higher total surface area, which in turn improved the release rate.^42^ A greater HLB value would also enable SDC to better solubilize the drug in the external aqueous phase, leading to noticeably (*P* < 0.0001) larger release profiles than T80.^43^

 In comparison to T80 stabilized particles, SDC generated significantly (*P* < 0.0001) larger negative ZP values. Because it is deposited on the particle/water interface and creates an electric double layer following ionization in water, SDC is an anionic emulsifier that raises ZP.^44^ Furthermore, SDC molecules stabilize the system by electrostatic repulsion and are held up more tightly around the particle and consequently have a lesser effect on the thickness of the diffuse layer, retaining higher ZP, whereas non-ionic surfactants (T80) have a more profound effect on extending the diffuse layer, which generates lower ZP.^45^ It is worth noting that although the T80 could not ionize into a charged group like SDC, it demonstrated reasonably high negative ZP values, the reason might be due to molecular polarization and the adsorption of T80 on the particle/water interface and electric double layer similar to anionic SAA was formed.^46^

###  Selection of the Optimal Formulation

 The goal of pharmaceutical formulation optimization is to reduce the number of variables required to produce a high-quality product. Design Expert^®^ software (Stat-Ease, Inc., Minneapolis, USA) was used to create specific criteria for choosing the optimum formula. Particles having the highest EE%, ZP as an absolute value, Q6h, and the lowest PS and PDI are preferred by these criteria. Analysis of variance (ANOVA) was used to ascertain the significance of each element after the experimental findings were analyzed using Design Expert^®^ software to independently identify the main effects of these factors. The optimal formula from experimental design, which met the criteria determined before, was PNLC16, which is composed of GTS as solid lipid and labrafil as liquid lipid, at solid lipid: liquid lipid ratio of 50:50% and is stabilized by SDC. Our experiment was validated by comparing the optimal formula’s predicted and observed responses, as indicated in Table 3. The observed and projected values showed a strong connection. PNLC16 was subsequently determined to be the best formula for the upcoming evaluations.

###  Transmission Electron Microscopy (TEM)

 The particles’ unique and spherical form was shown by TEM examination (Figure 3A). There were no anisometric-shaped particles in sight. The PS determined by dynamic light scattering and the PS from TEM examination agreed well.

**Figure 3:**
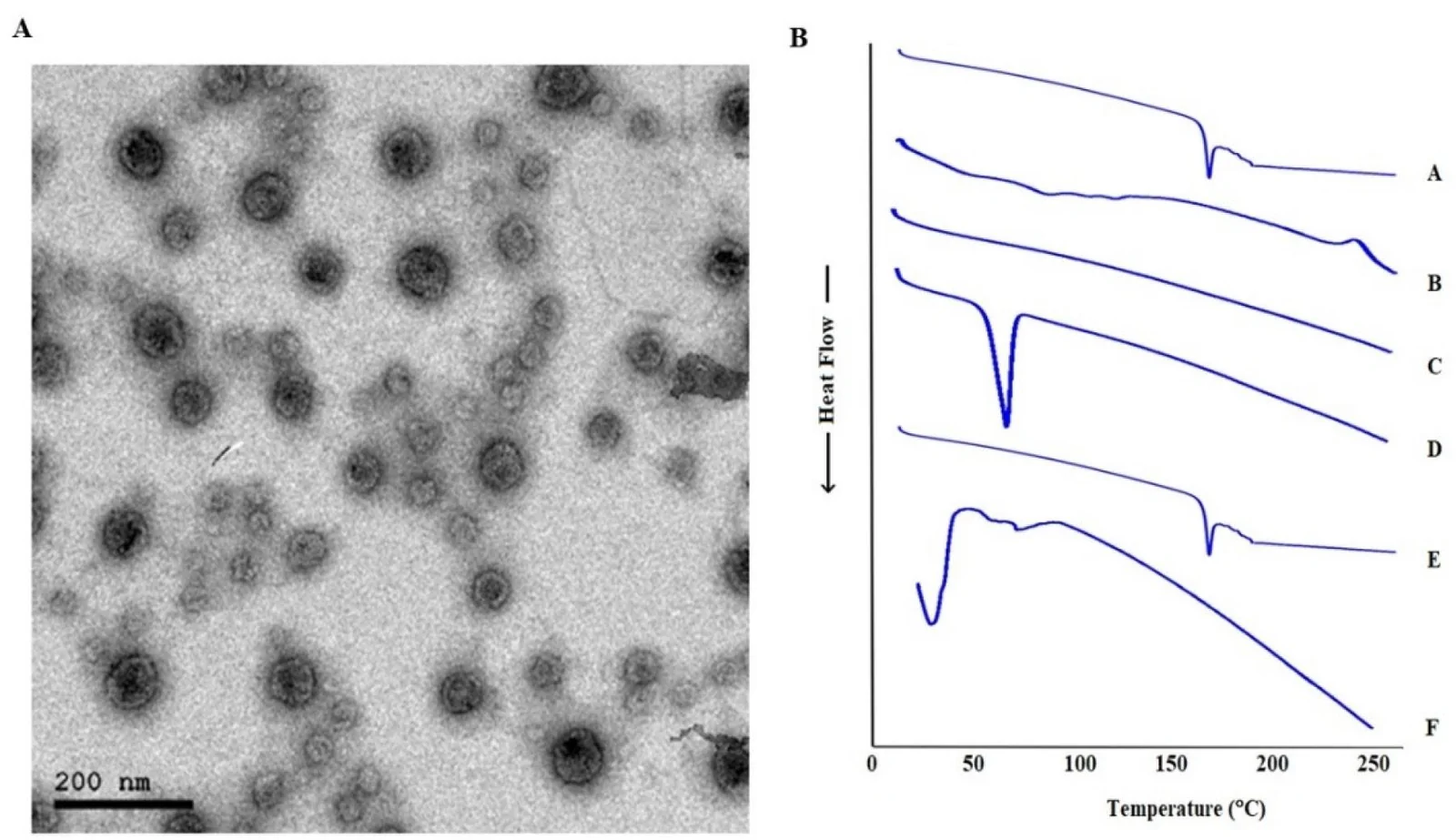
A) Transmission electron micrograph of the optimum PEGylated nanostructured lipid carriers (PNLC16) B) DSC thermograms of (A) OLM, (B) SDC, (C) Labrafil, (D) GTS, (E) physical mixture of PNLC16 components, and (F) PNLC16

###  Differential Scanning Calorimetry (DSC)

 As clarified in Figure 3B, OLM manifested a melting endothermic peak at 184.14°C.^2^ SDC’s thermogram showed a broad endotherm that began at 110.82°C, most likely as a result of water molecule loss, and ended at 214°C with an exothermic recrystallization peak.^47^ Aubert-Pouëssel et al. reported that labrafil has endothermal peaks at -80/-65/-47/-24/-1℃ and exothermal peaks at -74/-60/-40℃.^48^ However, because labrafil was liquid at room temperature, a DSC trace could not be recorded under the experimental conditions used. At 51°C, the GTS thermogram showed a significant melting peak.^49^ The OLM characteristic peak was visible in the DSC of the physical mixture of OLM and PNLC components, confirming that the OLM remained crystalline. The melting peak for OLM was not visible on the PNLC16 thermogram. This demonstrated that OLM had been effectively confined inside the particles.^13^

###  Stability Study 

 Formulations of nanoparticles have the potential to agglomerate during storage, which can alter PS, PDI, and ZP. Drug leaking from the particles and a decrease in the EE% could be the outcomes of these modifications. Visual inspection was used to check for particle aggregation and appearance alternation. There has been no evidence of gel formation or phase separation. The PS, PDI, and Q6h of the stored PNLCs preparation (PNLC16) at 4°C and 25°C after 45 and 90 days did not change significantly from the newly created one, according to statistical analysis (Table 4). Furthermore, EE% measurements remained constant, indicating that there was no drug expulsion from the formula during the trial time. The storage stability of colloidal dispersion systems is strongly influenced by their ZP. PNLC16’s negative charge was increased by the presence of SDC. The negative surface charge of -44.40 ± 5.38 mV as shown in Table 4 indicates greater repulsion between the particles thus particle aggregation is less likely to occur, which assured the stability potential of the developed PNLC16. The neutral PEG in PNLCs’ structure, which stabilized particles by steric hindrance, is likely also responsible for the particles’ exceptional stability in aqueous solution.^50^ Additionally, thick and elastic interfacial films with high MW of GTS are often more stress-resistant than those with low MW solid lipids, which are more likely to coalesce when pressed into close proximity.^51^ The PNLC16’s stability under storage conditions was validated by the earlier findings.

**Table 4:** stability study results for PNLC 16 after 45 and 90 days at 4 ℃ and 25 ℃.

	**Day**	**PNLC 16 4℃**	**PNLC 16 25℃**
EE%	0	39.68 ± 1.90	39.68 ± 1.90
	45	35.30 ± 5.32	34.04 ± 0.70
	90	35.16 ± 1.65	37.77 ± 0.47
PS(nm)	0	374.50 ± 39.5	374.50 ± 39.50
	45	386.45 ± 2.7	380.95 ± 5.1
	90	390.9 ± 108.6	393 ± 50.9
PDI	0	0.04 ± 0.01	0.04 ± 0.01
	45	0.05 ± 0.02	0.05 ± 0.12
	90	0.051 ± 0.001	0.05 ± 0.01
ZP(mV)	0	-44.40 ± 5.38	-44.40 ± 5.38
	45	-40.65 ± 7.79	-38.9 ± 7.75
	90	-39.50 ± 11	-39.0 ± 8.5
Q6h (%)	0	48.48 ± 1.17	48.48 ± 1.17
	45	49.72 ± 1.13	47.9 ± 1.3
	90	51.832 ± 1.17	50.32 ± 1.28

Abbreviations: PNLC, PEGylated nanostructured lipid carriers; EE%, entrapment efficiency percentage; PS, particle size; PDI, polydispersity index; ZP, Zeta potential, and Q6h, Amount of drug released after 6h.

###  Ex-Vivo Studies

####  Comparative Study Between Ex-Vivo Permeation Using Shed Snake Skin and Rat Skin 

 A wide range of animal models has been suggested as a suitable replacement for human skin and has been used to evaluate percutaneous permeation of drugs. The *ex vivo* permeation study was performed using two skin models: rat skin and shed snake skin. Rat skin is a full-thickness skin that consists of SC with underlying tissues of viable epidermis and dermis. Shed snake skin is a nonliving tissue which can be obtained without sacrifices. It consists of three layers – beta, meso, and alpha layers – and is composed of two very different regions – scales and separating these, hinges; the scales are firm, while the hinges are soft.^13^ The most representative model for estimating a drug’s transdermal penetration is human skin*. Ex-vivo* evaluation of percutaneous penetration has been performed on skin from a variety of sources, including cosmetic surgery and amputations. But because of its limited availability, animal skin is typically utilized. Numerous animal models have been proposed as a practical substitute for human skin and have been employed to gauge the percutaneous penetration of medications. These include models of primates, guinea pigs, mice, rats, pigs, and snakes.^52^ Comparing the data in the field of transdermal medication administration is extremely challenging, though, due to the large number of animal species that have been documented in the literature. The data can be greatly manipulated by the variety of techniques used with a particular skin model, including diffusion cell type, skin temperature, receiver media, application dose, and diffusion area. To choose the best representative model for human skin, it is necessary to screen several animal skins. The *ex-vivo* permeation was carried out via two skin models, namely, shed snake skin and rat skin. Shed snake skin is composed of three layers: beta, meso and alpha layers.^53^ Shed snake skin was donated by Giza Zoo, Giza, Egypt. Rat skin, however, is made of SC and is full thickness, with functioning epidermis and dermis layers underneath.^54^ Both membranes were shown to penetrate OLM in the same sequence of PNLC16 > OLM suspension (Figure 4). The significantly lower permeability of OLM suspension than PNLC16 may be attributed to the characteristics of the drug. It is thought that the low OLM penetration may be caused by the high log P value of 5.6.^2^ It has previously been documented that the skin permeability and lipophilicity of different medicines exhibit a distinctive parabolic relationship, with the highest permeability at log P of roughly three to four.^55^ As a result, for highly hydrophobic medications like OLM, the pace at which they penetrate the skin is determined by their passage through and subsequent clearance from the SC. Notably, the permeability parameters of shed snake skin were numerically (albeit not substantially) greater, with permeation fluxes of 12.19 ± 0.72 and 66.36 ± 3.56 (μg/cm2/h) for OLM and PNLC16, respectively, compared to 10.32 ± 1.10 and 49.88 ± 18.12 (μg/cm2/h) from rat skin (Table 5). This could be because rat skin is thicker than shed snake skin, with the former being thicker than the latter. Because it solely represents the barrier, the shed snake skin is only about 0.02-0.03 mm thick, but the typical thickness of hairless rat skin is 0.70-0.86 mm because it includes both the barrier and the underlying living tissues.^13^

**Figure 4:**
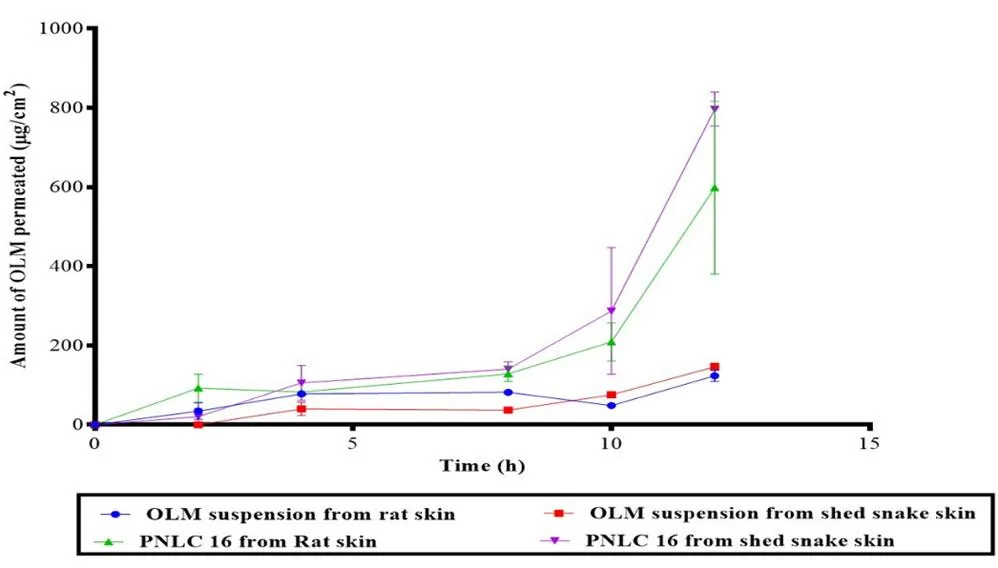
Cumulative amount of OLM permeated per unit area across excised rat skin and shed snake skin from PNLC16 relative to drug suspension

**Table 5:** Influence of OLM suspension and PNLC16 on mean Blood pressure in MPA-induced hypertensive rats

**Time (h)**	**Negative Control** **(mmHg)**	**HT control** **(mmHg)**	**Market tablet** **(mmHg)**	**PNLC16** **(mmHg)**	**% Reduction in BP** **of market tablet**	**% Reduction in BP of PNLC16**
0	109.50 ± 3.50	171.00 ± 4.00	177.00 ± 3.26	161.33 ± 7.93	4.00 ± 2.00%	6.00% ± 5.00%
1	126.20 ± 1.20	187.50 ± 2.50	138.00 ± 0.81	128.00 ± 8.60	26.00 ± 0%	32.00% ± 6.00%
2	129.00 ± 1.00	166.50 ± 6.50	127.20 ± 1.83	129.33 ± 8.99	24.00 ± 1.00%	22.00% ± 7.00%
4	126.70 ± 2.70	168.00 ± 8.00	128.90 ± 0.83	135.66 ± 6.12	23.00 ± 1.00%	19.00% ± 4.00%
6	117.70 ± 4.20	166.00 ± 4.00	126.50 ± 6.94	122.00 ± 8.00	24.00 ± 4.00%	27.00% ± 7.00%
8	114.70 ± 1.70	169.50 ± 2.50	140.10 ± 6.40	115.50 ± 6.50	17.00 ± 4.00%	32.00% ± 5.00%
24	117.70 ± 0.70	185.50 ± 5.50	165.00 ± 8.16	127.50 ± 2.50	11.00 ± 4.00%	31.00% ± 2.00%

BP: Blood pressure, PNLC: PEGylated nanostructured lipid carrier. Data represented as mean ± SD (n = 6).

###  In-Vivo Studies 

 All experimental animal studies were approved by the ethical committee of the Faculty of Pharmacy, Cairo University (reference number = (PI) 1867). The animals were subjected to anesthesia (using ketamine (200 mg/kg) and xylazine (20 mg/kg)) followed by decapitation.^13^

###  Histopathological Study

 The purpose of this investigation was to identify any potential irritation that might result from the permeation enhancers used in PNLC formulation. The untreated rat skin control (group I) photograph in Figure 5 displayed normal condition with distinct skin characteristics. Furthermore, there were no visible indications of inflammation in the skin sample treated with OLM suspension (group II) and PNLC16 (group III) formulation. The tolerability of PNLC16 towards skin was validated by earlier data.

**Figure 5:**
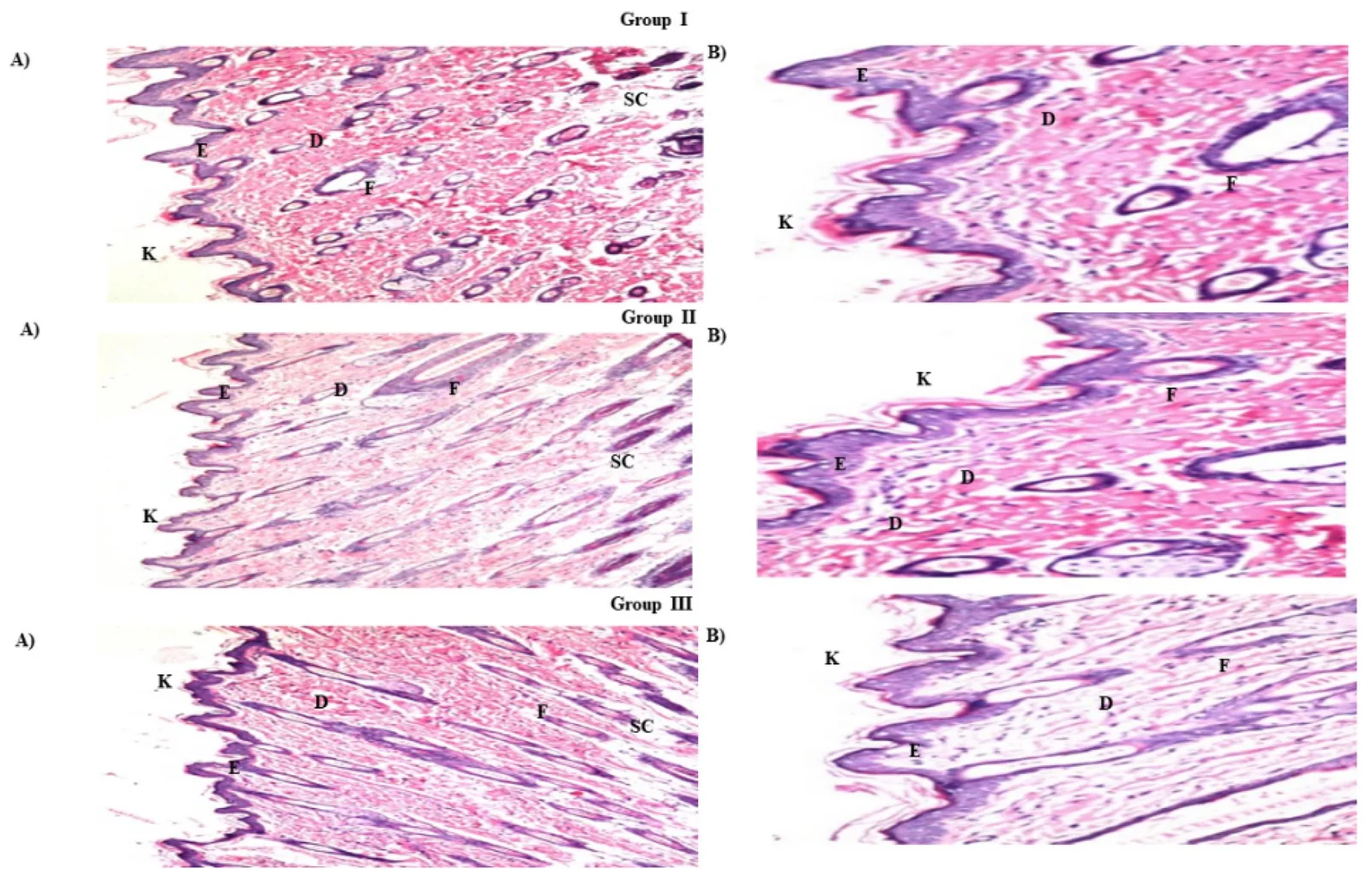
Photomicrographs showing histopathological sections (hematoxylin and eosin stained) of normal untreated rat skin (group I), rat skin treated with OLM suspension (group II) and rat skin treated with PNLC16 (group III). (A) – (B) denote the magnification power of 16 x to illustrate all skin layers and 40x to identify the epidermis and dermis. Abbreviations: K, keratin; E, epidermis; D, dermis; F, hair follicles and SC, subcutaneous fat

###  Antihypertensive Study 

 OLM is an ester prodrug of olmesartan. Subsequently, in assessing the transdermal bioavailability of OLM from PNLC 16 it is speculated to be converted in the blood circulation to Olmesartan due to the presence of esterase enzymes.By tracking the reduction in blood pressure after applying the ideal formulation to the skin of hypertensive rats, the antihypertensive effect of transdermaly applied PNLC16 was evaluated. Administration of MPA increased the BP above 150 mmHg in normotensive rats. After one hour of treatment, transdermally applied PNLC16 was able to return the blood pressure of MPA-induced hypertensive rats to normal levels. The greatest drop, 32.00 ± 5.00%, occurred at the 8-hour mark, and the blood pressure remained under control for up to 26 hours. On the other hand, two hours after oral tablet delivery, the blood pressure of the treated group returned to normal, and it remained under control for a maximum of six hours. The PNLC16 treated group’s blood pressure values at 28 and 48 hours were not normal, just like those of the oral tablet group, but there was a significant difference (*P* < 0.05) between the two groups. The PNLC16 treated group’s BP values decreased by 18.00 ± 2.00% and 11.00 ± 2.00%, respectively, compared to 1.00 ± 0.00% and 3.00 ± 1.00% for the oral tablet and PNLC16 treated groups at 28 and 48 hours. Additionally, post hoc analysis revealed that, at 8 and 24 hours after treatment, there was a significant difference (*P* ≤ 0.05) between the groups treated with oral tablets and the normal control group, but not between the PNLC16-treated group and the normal control group. These findings indicate that the developed particles were effective in returning the rat BP of MPA-induced hypertensive rats to normal values for prolonged period compared to oral tablets.

## Conclusion

 With a log P of 5.6, OLM is a very hydrophobic substance, which may be the cause of its poor transdermal penetration efficacy. PNLCs were selected as a drug transdermal permeability augmentation carrier in order to address this issue. Using a 2^4^ complete factorial design, PNLCs were created using hot melt emulsification in conjunction with high-speed stirring and ultrasonication. The study suggested the importance of controlling the independent variables during the formulation process as they greatly influenced EE%, PS, PDI, ZP, and Q6h. DSC tests on PNLC16 verified that OLM was trapped inside the structure of PNLCs. Furthermore, PNLC16 outperformed drug suspension through shed rat and snake skins, according to *ex-vivo* permeation experiments. Additionally, a histological investigation verified that PNLC16 does not irritate when applied to the skin of rats. Additionally, PNLC16 showed superiority over market tablets in a pharmacodynamic study and was able to maintain BP at normal levels for prolonged time. Based on the results obtained from this study PNLC16 could be considered as a promising formulation for transdermal delivery of OLM, in order to improve its bioavailability and sustain its effect which might improve patient compliance and reduce cost of the therapy. Further, a comprehensive pharmacokinetic profiling will be pursued in forthcoming studies to strengthen translational relevance.

## Competing Interests

 The authors declare that they have no competing interests.

## Ethical Approval

 Not applicable.
